# 3,5-Bis(2,4-dinitro­phen­yl)-4-nitro-1*H*-pyrazole acetone monosolvate

**DOI:** 10.1107/S1600536812001146

**Published:** 2012-01-14

**Authors:** Logesh Mathivathanan

**Affiliations:** a Deparment of Chemistry, University of Puerto Rico – Rio Piedras, San Juan, PR 00936, USA

## Abstract

The title structure, C_15_H_7_N_7_O_10_·C_3_H_6_O, was prepared by penta­nitration of 3,5-diphenyl-1*H*-pyrazole. The proton attached to a pyrazole N atom forms a hydrogen bond with the O atom of the acetone solvent mol­ecule, owing to the NO_2_ enhanced acidity of the proton. The NO_2_ group on the phenyl C atom is twisted by 33.9 (2)° from coplanarity with the ring in order to avoid a short intra­molecular O⋯O contact with an O atom of an adjacent pyrazole-bonded NO_2_ group.

## Related literature

For the nitration of 1*H*-pyrazole, see: Maresca *et al.* (1997 ▶). For the crystal structure of 3,5-diphenyl-1*H*-pyrazole, which shows a hydrogen-bonded tetra­meric structure, see: Raptis *et al.* (1993 ▶). For a crystallographic and *ab initio* study of 1*H*-pyrazoles, see: Foces-Foces *et al.* (2000 ▶).
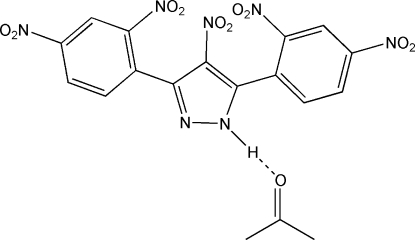


## Experimental

### 

#### Crystal data

C_15_H_7_N_7_O_10_·C_3_H_6_O
*M* *_r_* = 503.35
Monoclinic, 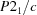

*a* = 14.886 (10) Å
*b* = 7.678 (5) Å
*c* = 19.801 (13) Å
β = 104.944 (9)°
*V* = 2187 (2) Å^3^
*Z* = 4
Mo *K*α radiation
μ = 0.13 mm^−1^
*T* = 298 K
0.31 × 0.19 × 0.18 mm

#### Data collection

Bruker SMART 1K CCD diffractometer
Absorption correction: multi-scan (*SADABS*; Bruker, 2005 ▶) *T*_min_ = 0.836, *T*_max_ = 0.977
15182 measured reflections
5041 independent reflections
2703 reflections with *I* > 2σ(*I*)
*R*_int_ = 0.068

#### Refinement

*R*[*F* ^2^ > 2σ(*F* ^2^)] = 0.064
*wR*(*F* ^2^) = 0.202
*S* = 1.03
5041 reflections
332 parameters
H atoms treated by a mixture of independent and constrained refinement
Δρ_max_ = 0.27 e Å^−3^
Δρ_min_ = −0.27 e Å^−3^

### 

Data collection: *SMART* (Bruker, 2005 ▶); cell refinement: *SAINT* (Bruker, 2005 ▶); data reduction: *SAINT*; program(s) used to solve structure: *SHELXS97* (Sheldrick, 2008 ▶); program(s) used to refine structure: *SHELXL97* (Sheldrick, 2008 ▶); molecular graphics: *SHELXTL* (Sheldrick, 2008 ▶); software used to prepare material for publication: *SHELXTL*.

## Supplementary Material

Crystal structure: contains datablock(s) I, global. DOI: 10.1107/S1600536812001146/qk2027sup1.cif

Structure factors: contains datablock(s) I. DOI: 10.1107/S1600536812001146/qk2027Isup2.hkl

Supplementary material file. DOI: 10.1107/S1600536812001146/qk2027Isup3.cdx

Supplementary material file. DOI: 10.1107/S1600536812001146/qk2027Isup4.cml

Additional supplementary materials:  crystallographic information; 3D view; checkCIF report

## Figures and Tables

**Table 1:** Hydrogen-bond geometry (Å, °)

*D*—H⋯*A*	*D*—H	H⋯*A*	*D*⋯*A*	*D*—H⋯*A*
N2—H2*A*⋯O11^i^	0.92 (3)	1.87 (4)	2.786 (4)	177 (3)

Symmetry code: (i) 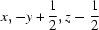.

**Fig. 1.**
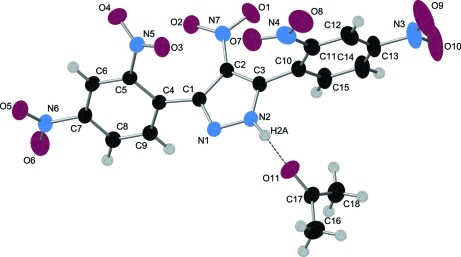
Molecular structure of the title compound (I) showing 30% thermal ellipsoids and atom labeling scheme. The symmetry transformation for the shown acetone molecule is x, 1/2-y, z-1/2.

## supplementary crystallographic information

### Comment

Aromatic electrophilic nitration (Maresca *et al.*, 1997) of 3,5-diphenyl-1*H*-pyrazole using nitration mixture (H_2_SO_4_/HNO_3_) produces the title compound, 3,5-bis-(2,4-dinitrophenyl)-4-nitro-1*H*-pyrazole. The crystal structure of the parent 3,5-diphenyl-1*H*-pyrazole is a H-bonded tetrameric structure (Raptis *et al.*, 1993), while the title compound (I) is not. Poly-nitration, in addition to a H-bonding to the solvent acetone molecule prevents tetramer formation in (I) (Fig. 1). H-bonding induced supramolecular network formation in 1*H*-pyrazoles has been summarized by Foces-Foces *et al.* (2000).

An intermolecular dipole-dipole interaction makes N4 and O2(1-*x*, *y*-1/2, 1/2-*z*) lie at 2.89 Å. In (I), all the NO_2_-groups are coplanar or nearly coplanar to their carrier rings, except the one attached to C5, which is twisted by 33.9 (2) relative to the phenyl ring in order to avoid a short intramolecular contact with O2.

### Experimental

For a general procedure for nitration of pyrazole, see Maresca *et al.* (1997). A flask containing 5 ml of conc. H_2_SO_4_ and 5 ml HNO_3_ kept in an ice bath was charged with 3,5-diphenyl-1*H*-pyrazole (97%, Aldrich; 2.0 g, 9.07 mmol) followed by the addition of 10 ml of H_2_SO_4_. The mixture was heated at 110° C for 2 days. After conventional neutralization and extractions, several colourless crystals of the title compound (I) were obtained from acetone.

### Refinement

All non-hydrogen atoms were refined anisotropically. Most H-atoms were positioned geometrically (C—H = 0.93 and 0.96 Å) and treated as riding (*U*_iso_(H) = 1.2*U*_eq_(C)). The pyrazole nitrogen H2A was located in a difference Fourier map and was then fully refined.

### Crystal data

**Table:**

C_15_H_7_N_7_O_10_·C_3_H_6_O	*F*(000) = 1032
*M**_r_* = 503.35	*D*_x_ = 1.529 Mg m^−^^3^
Monoclinic, *P*2_1_/*c*	Mo *K*α radiation, λ = 0.71073 Å
Hall symbol: -P 2ybc	Cell parameters from 5811 reflections
*a* = 14.886 (10) Å	θ = 2.1–27.1°
*b* = 7.678 (5) Å	µ = 0.13 mm^−^^1^
*c* = 19.801 (13) Å	*T* = 298 K
β = 104.944 (9)°	Polygon, colourless
*V* = 2187 (2) Å^3^	0.31 × 0.19 × 0.18 mm
*Z* = 4	

### Data collection

**Table:**

Bruker SMART 1K CCD diffractometer	5041 independent reflections
Radiation source: fine-focus sealed tube	2703 reflections with *I* > 2σ(*I*)
graphite	*R*_int_ = 0.068
φ and ω scans	θ_max_ = 28.0°, θ_min_ = 2.1°
Absorption correction: multi-scan (*SADABS*; Bruker, 2005)	*h* = −19→18
*T*_min_ = 0.836, *T*_max_ = 0.977	*k* = −10→8
15182 measured reflections	*l* = −25→25

### Refinement

**Table:**

Refinement on *F*^2^	Secondary atom site location: difference Fourier map
Least-squares matrix: full	Hydrogen site location: inferred from neighbouring sites
*R*[*F*^2^ > 2σ(*F*^2^)] = 0.064	H atoms treated by a mixture of independent and constrained refinement
*wR*(*F*^2^) = 0.202	*w* = 1/[σ^2^(*F*_o_^2^) + (0.0696*P*)^2^ + 1.6838*P*] where *P* = (*F*_o_^2^ + 2*F*_c_^2^)/3
*S* = 1.03	(Δ/σ)_max_ = 0.005
5041 reflections	Δρ_max_ = 0.27 e Å^−^^3^
332 parameters	Δρ_min_ = −0.27 e Å^−^^3^
0 restraints	Extinction correction: *SHELXL97* (Sheldrick, 2008), Fc^*^=kFc[1+0.001xFc^2^λ^3^/sin(2θ)]^-1/4^
Primary atom site location: structure-invariant direct methods	Extinction coefficient: 0.0141 (17)

### Special details

**Table:**

Geometry. All e.s.d.'s (except the e.s.d. in the dihedral angle between two l.s. planes) are estimated using the full covariance matrix. The cell e.s.d.'s are taken into account individually in the estimation of e.s.d.'s in distances, angles and torsion angles; correlations between e.s.d.'s in cell parameters are only used when they are defined by crystal symmetry. An approximate (isotropic) treatment of cell e.s.d.'s is used for estimating e.s.d.'s involving l.s. planes.
Refinement. Refinement of *F*^2^ against ALL reflections. The weighted *R*-factor *wR* and goodness of fit *S* are based on *F*^2^, conventional *R*-factors *R* are based on *F*, with *F* set to zero for negative *F*^2^. The threshold expression of *F*^2^ > σ(*F*^2^) is used only for calculating *R*-factors(gt) *etc*. and is not relevant to the choice of reflections for refinement. *R*-factors based on *F*^2^ are statistically about twice as large as those based on *F*, and *R*– factors based on ALL data will be even larger.

### Fractional atomic coordinates and isotropic or equivalent isotropic displacement parameters (Å^2^)

**Table:**

	*x*	*y*	*z*	*U*_iso_*/*U*_eq_	
O1	0.47956 (18)	−0.0956 (4)	0.26917 (13)	0.0968 (10)	
O2	0.59247 (15)	−0.0052 (4)	0.22802 (11)	0.0746 (7)	
O3	0.55406 (17)	−0.1803 (3)	0.08688 (13)	0.0730 (7)	
O4	0.70324 (18)	−0.1641 (3)	0.12926 (15)	0.0868 (8)	
O5	0.82468 (17)	0.3068 (4)	0.00361 (16)	0.0910 (9)	
O6	0.7904 (2)	0.5619 (4)	0.03522 (18)	0.1034 (10)	
O7	0.3592 (2)	−0.3312 (4)	0.12047 (15)	0.0919 (8)	
O8	0.2556 (2)	−0.5123 (4)	0.13596 (16)	0.1052 (10)	
O9	0.0670 (4)	−0.3824 (9)	0.2888 (3)	0.205 (3)	
O10	0.0655 (3)	−0.1292 (9)	0.3325 (2)	0.187 (3)	
O11	0.13846 (15)	0.4236 (3)	0.49406 (14)	0.0766 (7)	
N1	0.38256 (16)	0.1537 (3)	0.05753 (13)	0.0565 (6)	
N2	0.31564 (17)	0.0836 (4)	0.08520 (13)	0.0575 (7)	
N3	0.0923 (3)	−0.2335 (10)	0.2962 (2)	0.1284 (19)	
N4	0.2938 (2)	−0.3698 (4)	0.14388 (16)	0.0764 (8)	
N5	0.6270 (2)	−0.1003 (3)	0.10191 (14)	0.0612 (7)	
N6	0.77797 (18)	0.4055 (4)	0.02940 (16)	0.0678 (7)	
N7	0.50947 (19)	−0.0255 (4)	0.22360 (13)	0.0632 (7)	
C1	0.46197 (19)	0.1219 (4)	0.10473 (14)	0.0471 (6)	
C2	0.44426 (18)	0.0326 (4)	0.16222 (14)	0.0490 (7)	
C3	0.34937 (19)	0.0095 (4)	0.14748 (14)	0.0509 (7)	
C4	0.54864 (18)	0.1890 (4)	0.09021 (13)	0.0471 (6)	
C5	0.62536 (19)	0.0882 (4)	0.08719 (14)	0.0480 (6)	
C6	0.70118 (19)	0.1549 (4)	0.06789 (15)	0.0531 (7)	
H6	0.7512	0.0847	0.0656	0.064*	
C7	0.69967 (19)	0.3305 (4)	0.05218 (15)	0.0521 (7)	
C8	0.6277 (2)	0.4380 (4)	0.05695 (16)	0.0563 (7)	
H8	0.6295	0.5564	0.0474	0.068*	
C9	0.5529 (2)	0.3668 (4)	0.07611 (15)	0.0535 (7)	
H9	0.5041	0.4388	0.0798	0.064*	
C10	0.2870 (2)	−0.0633 (5)	0.18751 (15)	0.0578 (8)	
C11	0.2586 (2)	−0.2379 (5)	0.18530 (16)	0.0624 (8)	
C12	0.1955 (2)	−0.2936 (6)	0.22124 (18)	0.0798 (11)	
H12	0.1764	−0.4093	0.2193	0.096*	
C13	0.1618 (2)	−0.1736 (7)	0.25983 (19)	0.0853 (13)	
C14	0.1888 (3)	−0.0032 (7)	0.2649 (2)	0.0913 (13)	
H14	0.1658	0.0744	0.2924	0.110*	
C15	0.2510 (3)	0.0513 (6)	0.2283 (2)	0.0812 (11)	
H15	0.2694	0.1674	0.2310	0.097*	
C16	0.0075 (3)	0.2910 (6)	0.4196 (2)	0.0893 (12)	
H16A	0.0173	0.3761	0.3867	0.134*	
H16B	−0.0549	0.3008	0.4244	0.134*	
H16C	0.0168	0.1764	0.4032	0.134*	
C17	0.0743 (2)	0.3214 (4)	0.48844 (18)	0.0609 (8)	
C18	0.0603 (3)	0.2265 (6)	0.5498 (2)	0.0834 (11)	
H18A	0.0700	0.1042	0.5444	0.125*	
H18B	−0.0019	0.2454	0.5536	0.125*	
H18C	0.1038	0.2681	0.5913	0.125*	
H2A	0.256 (2)	0.082 (5)	0.0567 (18)	0.075 (11)*	

### Atomic displacement parameters (Å^2^)

**Table:**

	*U*^11^	*U*^22^	*U*^33^	*U*^12^	*U*^13^	*U*^23^
O1	0.0783 (17)	0.142 (3)	0.0701 (16)	−0.0068 (17)	0.0195 (13)	0.0425 (16)
O2	0.0506 (13)	0.108 (2)	0.0623 (13)	0.0001 (12)	0.0096 (10)	0.0091 (12)
O3	0.0783 (16)	0.0507 (13)	0.1029 (18)	−0.0055 (12)	0.0467 (14)	−0.0059 (12)
O4	0.0821 (17)	0.0677 (16)	0.114 (2)	0.0257 (14)	0.0310 (15)	0.0248 (14)
O5	0.0710 (16)	0.0891 (19)	0.131 (2)	0.0153 (14)	0.0578 (16)	0.0194 (17)
O6	0.100 (2)	0.0686 (18)	0.162 (3)	−0.0261 (16)	0.069 (2)	−0.0075 (18)
O7	0.108 (2)	0.0742 (18)	0.106 (2)	−0.0130 (16)	0.0497 (18)	−0.0134 (15)
O8	0.124 (2)	0.0762 (19)	0.103 (2)	−0.0379 (18)	0.0081 (18)	−0.0121 (15)
O9	0.189 (5)	0.263 (7)	0.197 (5)	−0.123 (5)	0.111 (4)	0.003 (4)
O10	0.159 (4)	0.299 (7)	0.145 (4)	−0.057 (4)	0.116 (3)	−0.024 (4)
O11	0.0555 (13)	0.0701 (15)	0.0989 (18)	−0.0122 (12)	0.0104 (12)	0.0034 (13)
N1	0.0487 (13)	0.0669 (16)	0.0573 (14)	0.0051 (12)	0.0197 (11)	0.0084 (12)
N2	0.0440 (13)	0.0720 (18)	0.0573 (14)	0.0031 (12)	0.0145 (12)	0.0073 (12)
N3	0.082 (3)	0.223 (6)	0.088 (3)	−0.044 (3)	0.036 (2)	0.024 (3)
N4	0.085 (2)	0.069 (2)	0.0689 (18)	−0.0151 (17)	0.0088 (16)	0.0013 (15)
N5	0.0655 (17)	0.0499 (15)	0.0749 (17)	0.0105 (14)	0.0305 (14)	0.0040 (12)
N6	0.0544 (15)	0.069 (2)	0.0849 (19)	−0.0030 (14)	0.0275 (14)	0.0056 (15)
N7	0.0565 (15)	0.0789 (19)	0.0539 (14)	−0.0019 (13)	0.0134 (12)	0.0097 (13)
C1	0.0482 (15)	0.0452 (15)	0.0502 (14)	0.0015 (12)	0.0168 (12)	−0.0008 (11)
C2	0.0481 (15)	0.0528 (17)	0.0472 (14)	0.0007 (12)	0.0143 (12)	0.0030 (12)
C3	0.0504 (16)	0.0543 (17)	0.0508 (15)	0.0001 (13)	0.0182 (12)	0.0013 (12)
C4	0.0461 (14)	0.0479 (15)	0.0485 (14)	0.0028 (12)	0.0140 (12)	−0.0001 (11)
C5	0.0493 (15)	0.0420 (15)	0.0543 (15)	0.0060 (12)	0.0163 (12)	0.0016 (12)
C6	0.0470 (15)	0.0532 (17)	0.0608 (17)	0.0090 (13)	0.0167 (13)	0.0000 (13)
C7	0.0443 (15)	0.0521 (17)	0.0617 (17)	−0.0024 (13)	0.0171 (13)	−0.0002 (13)
C8	0.0584 (17)	0.0437 (15)	0.0699 (18)	0.0006 (14)	0.0224 (15)	−0.0004 (13)
C9	0.0545 (16)	0.0461 (16)	0.0637 (17)	0.0062 (13)	0.0221 (14)	0.0014 (13)
C10	0.0495 (16)	0.070 (2)	0.0552 (16)	−0.0024 (15)	0.0162 (13)	0.0050 (14)
C11	0.0522 (17)	0.075 (2)	0.0559 (17)	−0.0110 (16)	0.0071 (14)	0.0053 (15)
C12	0.064 (2)	0.103 (3)	0.067 (2)	−0.030 (2)	0.0062 (17)	0.015 (2)
C13	0.058 (2)	0.140 (4)	0.062 (2)	−0.020 (2)	0.0228 (17)	0.011 (2)
C14	0.078 (3)	0.129 (4)	0.080 (3)	−0.006 (3)	0.044 (2)	−0.004 (2)
C15	0.079 (2)	0.093 (3)	0.085 (2)	−0.003 (2)	0.047 (2)	−0.007 (2)
C16	0.071 (2)	0.094 (3)	0.094 (3)	−0.009 (2)	0.006 (2)	−0.009 (2)
C17	0.0443 (16)	0.0538 (18)	0.084 (2)	0.0033 (14)	0.0152 (15)	−0.0014 (15)
C18	0.070 (2)	0.090 (3)	0.094 (3)	−0.002 (2)	0.028 (2)	0.006 (2)

### Geometric parameters (Å, °)

**Table:**

O1—N7	1.228 (3)	C4—C9	1.398 (4)
O2—N7	1.226 (3)	C5—C6	1.380 (4)
O3—N5	1.216 (3)	C6—C7	1.382 (4)
O4—N5	1.226 (3)	C6—H6	0.9300
O5—N6	1.225 (4)	C7—C8	1.375 (4)
O6—N6	1.216 (4)	C8—C9	1.378 (4)
O7—N4	1.218 (4)	C8—H8	0.9300
O8—N4	1.224 (4)	C9—H9	0.9300
O9—N3	1.200 (8)	C10—C15	1.391 (5)
O10—N3	1.209 (7)	C10—C11	1.402 (5)
O11—C17	1.219 (4)	C11—C12	1.385 (5)
N1—C1	1.327 (4)	C12—C13	1.372 (6)
N1—N2	1.365 (3)	C12—H12	0.9300
N2—C3	1.333 (4)	C13—C14	1.365 (6)
N2—H2A	0.92 (3)	C14—C15	1.380 (5)
N3—C13	1.480 (5)	C14—H14	0.9300
N4—C11	1.481 (5)	C15—H15	0.9300
N5—C5	1.476 (4)	C16—C17	1.484 (5)
N6—C7	1.471 (4)	C16—H16A	0.9600
N7—C2	1.418 (4)	C16—H16B	0.9600
C1—C2	1.411 (4)	C16—H16C	0.9600
C1—C4	1.485 (4)	C17—C18	1.477 (5)
C2—C3	1.378 (4)	C18—H18A	0.9600
C3—C10	1.478 (4)	C18—H18B	0.9600
C4—C5	1.394 (4)	C18—H18C	0.9600
			
C1—N1—N2	104.7 (2)	C7—C8—C9	118.7 (3)
C3—N2—N1	113.6 (2)	C7—C8—H8	120.7
C3—N2—H2A	130 (2)	C9—C8—H8	120.7
N1—N2—H2A	116 (2)	C8—C9—C4	121.6 (3)
O9—N3—O10	124.3 (5)	C8—C9—H9	119.2
O9—N3—C13	118.2 (6)	C4—C9—H9	119.2
O10—N3—C13	117.5 (6)	C15—C10—C11	117.7 (3)
O7—N4—O8	124.0 (4)	C15—C10—C3	117.4 (3)
O7—N4—C11	118.4 (3)	C11—C10—C3	124.8 (3)
O8—N4—C11	117.6 (3)	C12—C11—C10	121.2 (4)
O3—N5—O4	125.0 (3)	C12—C11—N4	117.2 (3)
O3—N5—C5	118.6 (3)	C10—C11—N4	121.6 (3)
O4—N5—C5	116.4 (3)	C13—C12—C11	118.2 (4)
O6—N6—O5	124.1 (3)	C13—C12—H12	120.9
O6—N6—C7	118.1 (3)	C11—C12—H12	120.9
O5—N6—C7	117.7 (3)	C14—C13—C12	122.9 (3)
O2—N7—O1	123.6 (3)	C14—C13—N3	119.5 (5)
O2—N7—C2	118.4 (2)	C12—C13—N3	117.7 (5)
O1—N7—C2	118.0 (3)	C13—C14—C15	118.4 (4)
N1—C1—C2	109.9 (2)	C13—C14—H14	120.8
N1—C1—C4	117.4 (2)	C15—C14—H14	120.8
C2—C1—C4	132.7 (2)	C14—C15—C10	121.6 (4)
C3—C2—C1	106.6 (2)	C14—C15—H15	119.2
C3—C2—N7	125.4 (3)	C10—C15—H15	119.2
C1—C2—N7	128.0 (3)	C17—C16—H16A	109.5
N2—C3—C2	105.2 (2)	C17—C16—H16B	109.5
N2—C3—C10	121.2 (3)	H16A—C16—H16B	109.5
C2—C3—C10	133.4 (3)	C17—C16—H16C	109.5
C5—C4—C9	117.0 (3)	H16A—C16—H16C	109.5
C5—C4—C1	125.3 (3)	H16B—C16—H16C	109.5
C9—C4—C1	117.7 (2)	O11—C17—C18	121.1 (3)
C6—C5—C4	122.9 (3)	O11—C17—C16	120.6 (3)
C6—C5—N5	116.5 (2)	C18—C17—C16	118.4 (3)
C4—C5—N5	120.6 (2)	C17—C18—H18A	109.5
C5—C6—C7	117.2 (3)	C17—C18—H18B	109.5
C5—C6—H6	121.4	H18A—C18—H18B	109.5
C7—C6—H6	121.4	C17—C18—H18C	109.5
C8—C7—C6	122.5 (3)	H18A—C18—H18C	109.5
C8—C7—N6	118.6 (3)	H18B—C18—H18C	109.5
C6—C7—N6	118.9 (3)		

### Hydrogen-bond geometry (Å, °)

**Table:**

*D*—H···*A*	*D*—H	H···*A*	*D*···*A*	*D*—H···*A*
N2—H2A···O11^i^	0.92 (3)	1.87 (4)	2.786 (4)	177 (3)

Symmetry codes: (i) *x*, −*y*+1/2, *z*−1/2.
